# FGF23 and Immune Cell Signatures Causally Linked to Subarachnoid Hemorrhage: Evidence From Multi‐Omics and Genetic Colocalization

**DOI:** 10.1002/brb3.71485

**Published:** 2026-05-08

**Authors:** Xingjie Shi, Cheng Zhang, Tao Yang, Zhiming Sun, Chao Wang, Lucheng Zhou, Shiqiang Hou, Ning Lin, Lanlan Zhang

**Affiliations:** ^1^ Department of Neurosurgery The Affiliated Chuzhou Hospital of Anhui Medical University Chuzhou China; ^2^ Department of Disinfection Supply Center The Affiliated Chuzhou Hospital of Anhui Medical University Chuzhou China; ^3^ Department of Science and Education Section The Affiliated Chuzhou Hospital of Anhui Medical University Chuzhou China

**Keywords:** subarachnoid hemorrhage, Mendelian randomization, inflammatory proteins, colocalization analysis

## Abstract

**Background:**

Inflammation and immune response significantly contribute to brain injury following subarachnoid hemorrhage (SAH), a severe neurological condition. This study employed Mendelian randomization, colocalization, and multi‐omics analysis to examine potential causal connections between inflammatory proteins, immune cells, and SAH, aiming to elucidate its pathogenesis.

**Methods:**

This study utilized publicly available data from genome‐wide association studies (GWAS), including protein QTL (pQTL) and RNA sequencing data. Bidirectional two‐sample Mendelian Randomization (MR) analysis was initially employed to evaluate the cause‐and‐effect relationships among inflammatory proteins, immune cells, and SAH. A comprehensive multi‐omics approach, encompassing transcriptome, colocalization, mediation MR, was employed to identify specific inflammatory proteins, immune cells, and potential drug targets.

**Results:**

A causal relationship between five inflammatory proteins and SAH was identified through MR analysis (CD6, FGF23, TGFB‐1, LIFR, and TGF‐α). Moreover, a causal relationship with SAH was identified in 22 types of immune cells. Subsequent multi‐omics analysis showed that FGF23 was a hub inflammatory protein, and its expression level was closely linked to the amount of CD4 Treg cells. Meta‐analysis and replication studies identified FGF23 as a risk factor for SAH, with a colocalization score of 0.74.

**Conclusion:**

This study successfully identified inflammatory proteins and immune cells associated with SAH, and revealed the complex genetic causality and drug targets of SAH.

## Graphical abstract

Causal links identified between five inflammatory proteins, 22 immune cell types, and subarachnoid hemorrhage (SAH) via Mendelian randomization. Multi‐omics integration (pQTL, eQTL) revealed regulatory mechanisms underlying inflammation in SAH. FGF23 and CD4 Tregs emerged as key therapeutic targets, supported by colocalization and replication analysis.

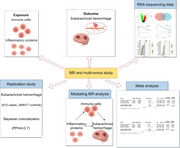

## 1 Introduction

Subarachnoid hemorrhage (SAH) is primarily attributed to the spontaneous rupture of intracranial aneurysms, leading to substantial increases in morbidity, mortality, and healthcare burden (Hoh et al. 2023). This condition predominantly affects adults around the age of 50 and has a profound influence on patients’ quality of life and public health (Claassen and Park 2022). A burst intracranial aneurysm causes blood to enter the subarachnoid space, triggering a series of inflammatory and immune responses. These pathological processes are intricately linked to the unfavorable prognosis observed in patients (Wu et al. 2021, Solár et al. 2022). Research indicates that brain injury following SAH can be alleviated through the modulation of microglial polarization and inflammatory responses (Shao et al. 2023, Li et al. 2018). Following subarachnoid hemorrhage (SAH), regulatory T cells (Tregs) become activated and regulate the immune response by inhibiting T cell proliferation and influencing the production of anti‐inflammatory factors (Sakaguchi et al. 2010). Furthermore, the release of inflammatory molecules from damaged neurons and necrotic cells into the extracellular milieu may exacerbate secondary injury after SAH (Sun et al. 2014). A thorough grasp of the immune‐inflammatory mechanisms linked to SAH and its outcomes is essential for improving patient treatment strategies.

Mendelian randomization (MR) has garnered significant attention in recent years. Genetic variants are utilized in this method as instrumental variables to deduce causal connections between exposure and outcome, minimizing confounding and reverse causality. As a result, MR improves the reliability of causal inference over traditional observational studies. MR provides stronger causal evidence than traditional observational studies. With the continuous update of GWAS data, there have been studies to examine the genetic association between immune‐inflammation and intracranial aneurysms (IAs) from gene expression and protein level expression.

In view of the lack of multi‐omics comprehensive analysis to examine the role of immune and inflammatory response targets in SAH. This study integrates multi‐omics and two‐sample MR analysis to identify inflammatory proteins and immune cells connected to SAH, explore their associations, and pinpoint potential drug targets, offering new insights and strategies for personalized SAH treatment.

## 2 Methods

### 2.1 Study Design and Data Source

**FIGURE 1:**
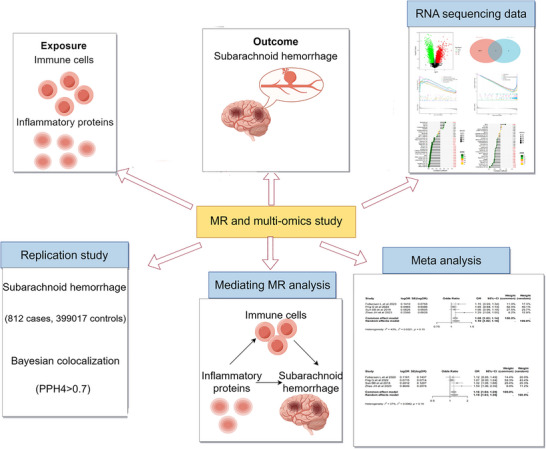
The flow chart of this study was made using FigDraw. The overall study framework illustrates data sources, Mendelian randomization analyses, colocalization, transcriptomic validation, and downstream integrative analyses.

This research made use of publicly accessible GWAS summary statistics and RNA sequencing data, thus not requiring additional ethical approval. Refer to Figure 1 for the study flow. The sample information is detailed as follows: 91 inflammatory proteins from Zhao et al. The study analyzed samples ranging from 11,778 to 14,744 (Zhao et al. 2023) and included 731 immune cells from Valeria Orru et al., with sample sizes between 1,244 and 3,659 (Orrù et al. 2020). The GWAS catalog served as the main source for additional pQTL data, encompassing GCST90012022, GCST90179299, and GCST90241167, with sample sizes from 2843 to 21,758. SAH data were derived from Sakaue et al. (2021), involving 1693 European ancestry cases and 471,562 controls of European ancestry. The replication study included 812 British ancestry cases and 399,017 British ancestry controls. This study utilized transcriptome sequencing datasets GSE36791 and GSE73378 from the GEO database. Dataset GSE36791 comprised 43 whole blood samples from SAH patients and 18 from healthy controls, while dataset GSE73378 included 103 patient and 107 control peripheral blood samples. A complete catalog of all datasets is provided in Table S1.

### 2.2 Recognition of Differentially Expressed Genes

The “limma” package detected significantly differentially expressed genes (DEGs) between the SAH and control groups, applying a significance threshold of *p* < 0.05 and logFoldChange (logFC) > 0.1. The “ggplot2” package was used to generate a volcano plot of DEGs.

### 2.3 Selection of Instrumental Variables

Immune cell and inflammatory protein‐related SNPs were identified applying significance thresholds of *p* < 5 × 10^−8^ and *p* < 5 × 10^−6^. Because only a few SNPs were screened, we set a significance level of *p* < 1 × 10^−5^ for the analyses that followed. To minimize linkage disequilibrium effects, we configured the parameter to 10,000 kb with *r* ^2^ = 0.001. SNPs with an F‐statistic exceeding 10 were chosen as IVs for additional analyses.

### 2.4 Analysis of Immune Infiltration

Single‐sample gene set enrichment analysis (ssGSEA) was employed to evaluate immune cell expression levels in datasets GSE36791 and GSE73378, and subsequent correlation examination was conducted to investigate the connection between gene expression and immune cell content. A correlation test *p*‐value below 0.05 indicates a relationship between inflammatory protein expression and immune cell content.

### 2.5 GSEA and Drug Target Enrichment Analysis

This study employed the GSEA method to determine if biological functions or signaling pathways linked to specific genes showed significant enrichment at the extremes of the gene expression ranking, suggesting a trend of up‐ or downregulation. We employed GSEA analysis to investigate functional or pathway activity differences between high‐ and low‐expression groups of inflammatory proteins. Data on genes and drugs were acquired from the Drug–Gene Interaction Database (DGIdb, https://dgidb.genome.wustl.edu/), and the “enrichplot” package was utilized to identify drugs linked to FGF23. Statistical significance was assigned to a *p*‐value under 0.05.

### 2.6 Meta‐Analysis and Bayesian Colocalization Analysis

We performed a meta‐analysis to further confirm the cause‐and‐effect link between inflammatory protein levels and SAH, utilizing exposure data from various studies. Replication studies validated the causal link between inflammatory proteins and SAH. Subsequently, we performed a colocalization analysis to explore SNPs potentially associated with inflammatory proteins and SAH. Previous research suggests that the following criteria should be used to screen these proteins: (1) H0: no cause‐and‐effect variant exists for either immune‐inflammation or SAH; (2) H1: a single cause‐and‐effect variant affects only gene expression; (3) H2: a single cause‐and‐effect variant influences only SAH risk; (4) H3: two distinct cause‐and‐effect variants affect the two traits separately; and (5) H4: a shared causal variant influences both traits (Giambartolomei et al. 2014). For causal variables linked to just two traits, FGF23 and SAH, the parameters are set as P1 = 1 × 10^−4^, P2 = 1 × 10^−4^, and P12 = 1 × 10^−5^. Finally, Colocalization is assumed when H4 (PPH4) exceeds 0.70.

### 2.7 Statistical Analysis

We applied FDR correction (FDR < 0.05) to results from the IVW method with *p*‐values below 0.05. In addition, to assess the consistency of the causal direction, we performed the MR‐Steiger test and reverse MR Analysis. To further explore potential biological pathways linking inflammatory proteins to SAH, we conducted a two‐step Mendelian randomization mediation analysis. In the first step, genetic instruments for inflammatory proteins were used to estimate their causal effects on immune cell traits. In the second step, immune cell traits were evaluated as exposures to assess their causal effects on SAH. The mediation proportion was calculated as the ratio of the indirect effect to the total effect, following a product‐of‐coefficients framework. Because this analysis was based on summary‐level data, mediation results are reported as point estimates and interpreted as exploratory. Statistical analyses, such as heterogeneity and pleiotropy tests, were carried out to determine the results' stability and reliability. Cochran's *Q* statistic was initially employed to evaluate heterogeneity among genetic variants in the IVW method. In cases where heterogeneity was detected, we switched to a random‐effects model for subsequent analyses. Next, we assessed horizontal pleiotropy by MR‐Egger regression (Burgess and Thompson 2017). The MR‐PRESSO approach is employed to tackle directional pleiotropy. The MR‐PRESSO method aims to detect and eliminate outlier single‐nucleotide polymorphisms (SNPs) potentially exhibiting horizontal pleiotropy. The overall test results by MR‐PRESSO showed that the existence of horizontal pleiotropy was statistically significant (Verbanck et al. 2018). Analyses utilized R software (v4.3.2) and packages such as “TwoSampleMR” (v0.5.6), “meta” (v7.0), “coloc” (v5.2.3), and “ggplot2” (v3.5.1).

## 3 Results

### 3.1 Causal Link between Inflammatory Proteins and SAH

**FIGURE 2:**
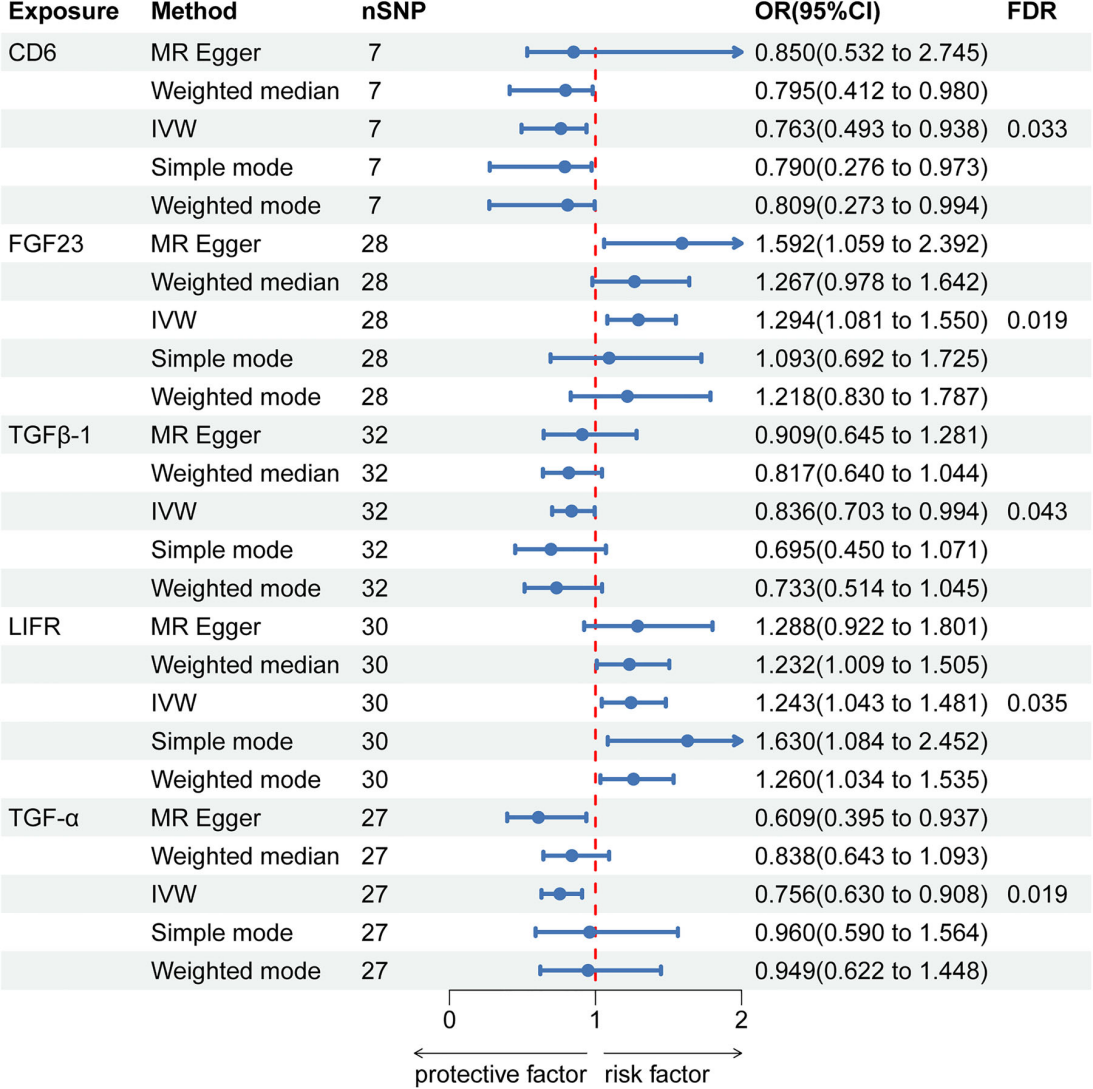
Forest plot estimates of the association between inflammatory proteins and risk of SAH. IVW, inverse variance weighted; OR, odds ratio; CI, confidence interval; FDR, false discovery rate.

In the forward MR analysis, five inflammatory proteins were identified as causal factors for SAH following correction (Figure 2). Including CD6, FGF23, TGFB‐1, LIFR, and TGF‐α. IVW analysis indicated a downward correlation between CD6 levels and SAH risk (OR = 0.68, 95% CI: 0.50–0.94, FDR < 0.05). SAH risk was linked positively with FGF23 levels (OR = 1.29, 95% CI: 1.08–1.55, FDR < 0.05). TGFB‐1 levels showed a negative correlation with SAH risk (OR 0.84, 95% CI 0.70–0.99, FDR < 0.05). Elevated LIFR levels were significantly linked to a higher risk of SAH (OR 1.24, 95% CI 1.04–1.48, FDR < 0.05). There was a downward relationship between TGF‐α levels and the risk of SAH (OR: 0.76, 95% CI, 0.63–0.91, FDR < 0.05). The findings indicate that lower levels of CD6, TGFB‐1, and TGF‐α correlate with a decreased risk of SAH, while higher levels of FGF23 and LIFR are linked to an increased SAH risk. Instrument strength was evaluated using F‐statistics for all genetic instruments. To maintain clarity, instrument‐strength visualization was provided for representative inflammatory protein exposures, while immune cell instruments were assessed numerically and filtered using standard thresholds (Figure S1). Sensitivity analysis revealed heterogeneity in SNPs associated with LIFR levels (*Q* = 50.17, *p* = 0.008). Therefore, the multiplicative random‐effects IVW model was applied instead of the fixed‐effect IVW estimator (Table S2). Reverse MR and Steiger test results did not reveal reverse causality (Table S3).

### 3.2 Causal Association Between Immune Cells and SAH

**FIGURE 3:**
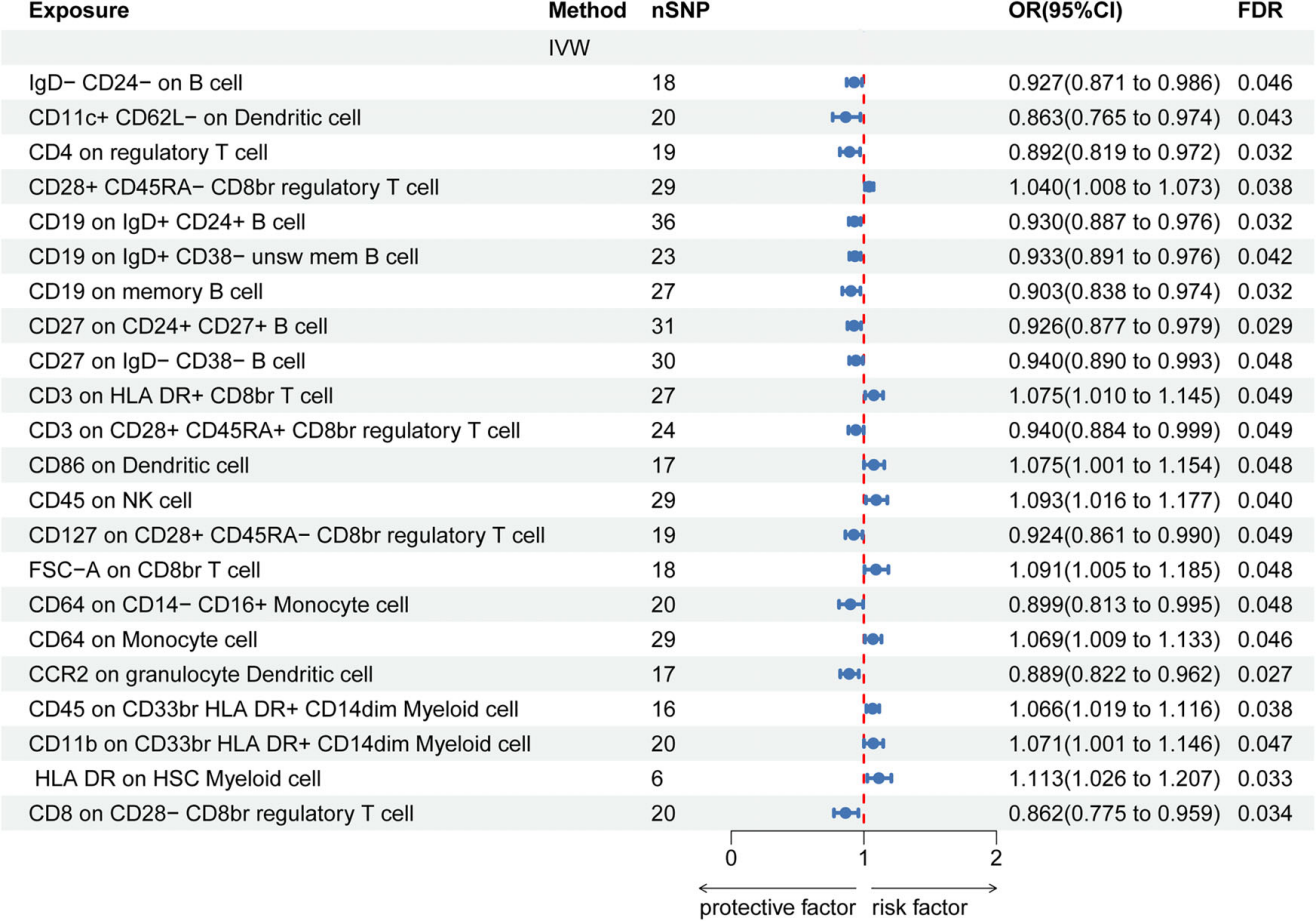
Forest plot estimates of the association between immune cells and risk of SAH. IVW, inverse variance weighted; OR, odds ratio; CI, confidence interval; FDR, false discovery rate.

Forward MR Analysis, as shown in Figure 3, showed that 22 immune cells were causally related to SAH after correction. The study investigates various immune cell markers, including IgD‐CD24− on B cells, CD11c+ CD62L− on dendritic cells, CD4 on regulatory T cells, CD28+ CD45RA− on CD8br regulatory T cells, and CD19 on both IgD+ CD24+ B cells and IgD+ CD38− unswitched memory B cells. It also examines CD19 on memory B cells, CD27 on CD24+ CD27+ B cells and IgD− CD38− B cells, CD3 on HLA DR+ CD8br T cells and CD28+ CD45RA+ CD8br regulatory T cells, CD86 on dendritic cells, CD45 on NK cells, CD127 on CD28+ CD45RA− CD8br regulatory T cells, FSC‐A on CD8br TBNK cells, CD64 on CD14− CD16+ monocytes and monocytes, CCR2 on granulocyte dendritic cells, CD45 and CD11b on CD33br HLA DR+ CD14dim myeloid cells, HLA DR on HSC myeloid cells, and CD8 on CD28− CD8br regulatory T cells. Among them, 10 were positively linked to SAH, and 12 were negatively linked to SAH (FDR < 0.05). Sensitivity analysis did not find significant heterogeneity in the results of 22 immune cells. The MR‐Egger *p*‐value indicating the effect of CD11b on CD33br HLA DR+ CD14dim myeloid cells was significant, as it was below 0.05. However, MR‐PRESSO was greater than 0.05 (Table S4). A per‐SNP summary including effect sizes and F‐statistics is provided in Table S5. Reverse MR analysis of immune cells using IVW identified a significant association between SAH and CD64 expression on CD14− CD16+ monocytes (FDR < 0.05) (Table S6). Steiger's result did not reveal a reverse association with supplementation (Table S6).

### 3.3 Identification of Differential Genes and Hub Inflammatory Proteins

**FIGURE 4:**
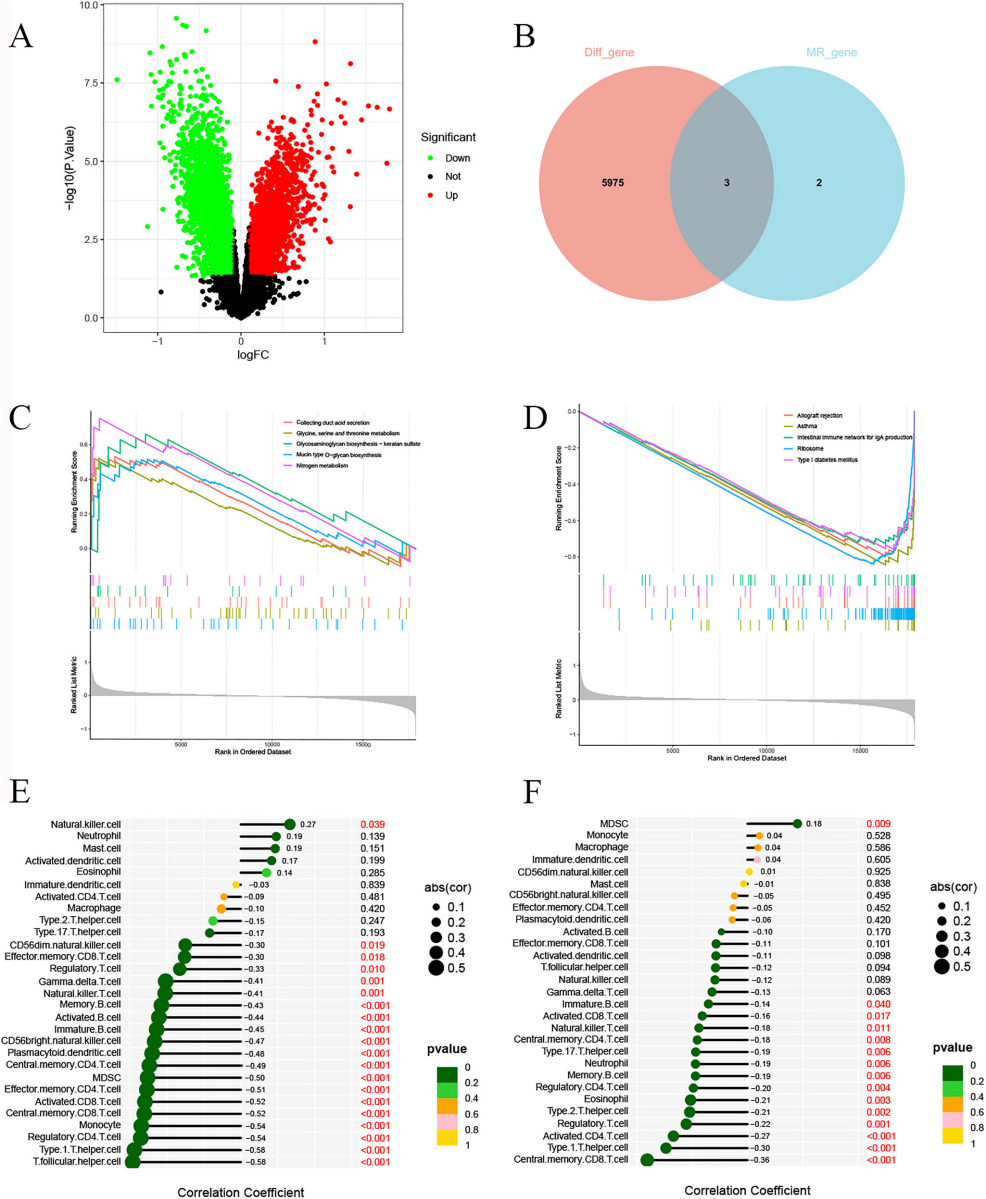
Identification and functional characterization of hub inflammatory proteins. (A) Volcano plot of differential genes. (B) Venn diagram of the intersection of differential genes and inflammatory proteins, MR results. GSEA enrichment analysis and immune cell infiltration. (C) The top 5 active biological functions in the FGF23 high‐expression group. (D) The top 5 active biological functions in the FGF23 low‐expression group. (E) Stick plot of correlation between FGF23 and a variety of immune cells in the GSE36791 dataset. (F) Stick plot of correlation between FGF23 and a variety of immune cells in the GSE73378 dataset.

We used the GSE36791 dataset for DEGs analysis in this research. The analysis indicates that a smaller p‐value enhances the trustworthiness of gene ranking and differential expression. After screening, we identified upregulation of 2537 DEGs and downregulation of 3441 DEGs (Table S7). The volcano plot highlights the upregulated and downregulated DEGs (Figure 4A). Furthermore, through Venn diagram analysis, the intersection analysis of inflammatory proteins obtained by MR Analysis and differential genes was performed (Figure 4B), and the key inflammatory proteins, FGF23 and TGF‐α, were identified.

### 3.4 Mediation Analysis of Hub Inflammatory Proteins

To explore the interaction between inflammatory proteins and SAH, we employed a two‐step method to evaluate the mediating effects of key identified inflammatory proteins. A significant correlation between inflammatory proteins and immune cells is essential for mediation. TGF‐α was not found to be significantly associated with any immune cells in our analysis. It indicates a causal link between FGF23 and CD4 regulatory T cells (CD4 Treg cells), implying that CD4 Treg cells may mediate the relationship between FGF23 and SAH, with mediating effects estimated at 10.8% (Table S8). This finding provides a new perspective to understand the role of FGF23 within the pathological process of SAH.

### 3.5 GSEA and Drug Enrichment Analysis

FGF23 was investigated for its potential impact on SAH through gene set enrichment analysis (GSEA). It indicates that FGF23 is primarily enriched in nitrogen metabolism, glycosaminoglycan biosynthesis‐keratan sulfate, and collecting duct acid secretion, based on the enrichment score (Figure 4C). Metabolism of glycine, serine, and threonine, along with mucin‐type O‐glycan biosynthesis and other biological processes. The downregulated expression was mainly linked to asthma, ribosome, and other biological processes (Figure 4D). Thirty drugs were associated with FGF23, including beta‐Glycerophosphoric acid, Cinacalcet, and alfacalcidol (*p* < 0.05) (Table S9).

### 3.6 Assessment of Immune Cell Infiltration

Mediation and enrichment analyses indicate that FGF23 is pivotal in immune‐inflammation processes. The ssGSEA algorithm was employed to examine immune cell profiles and the association between FGF23 and immune cell infiltration in SAH. The analysis of the GSE36791 dataset revealed a significant correlation between the expression of FGF23 and multiple immune cells (Figure 4E). Among them, CD4 Treg cells (cor = −0.54, *p* < 0.001) (Table S10). In the GSE73378 dataset, a significant association was also revealed (cor = −0.20, *p* = 0.004) (Figure 4F; Table S11).

### 3.7 Integrating Multi‐Omics Evidence Between Inflammatory Protein and SAH

**FIGURE 5:**
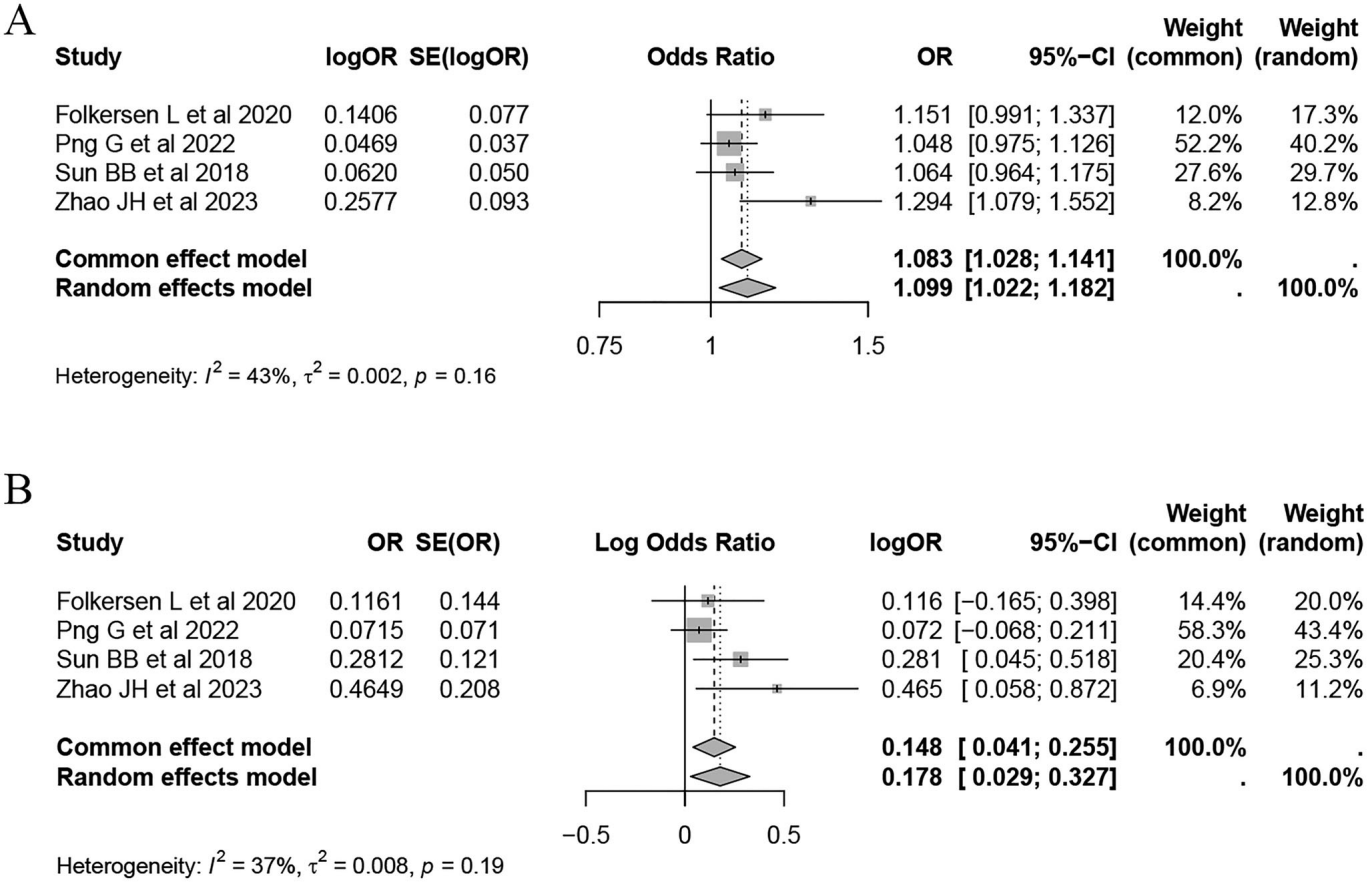
Meta‐analysis of the association between circulating FGF23 levels and SAH. (A) Meta‐analysis of the IVW method between four FGF23 protein level studies and SAH. (B) Meta‐analysis of the Egger method between four FGF23 protein level studies and SAH.

To determine the reliability of our conclusions, a meta‐analysis was performed, incorporating results from both MR and repeated MR analyses. The IVW and Egger meta‐analyses, supported by repeated MR analyses, indicate a possible causal relationship (OR_IVW_ = 1.08, 95% CI 1.03–1.14; OR_Egger_ = 1.16, 95% CI 1.04–1.29) (Figure 5A,B). In a replication study, we also confirmed that FGF23 enhanced the risk of SAH (OR = 1.61, 95%CI: 1.14–2.27) (Table S12). For FGF23, no genome‐wide significant cis‐pQTLs were identified in the pQTL dataset; therefore, the colocalization signal (PPH4 = 0.74) was driven by trans‐acting variants (Table S12). To visualize this shared genetic signal, we generated a GWAS–pQTL scatter plot illustrating the concordance of association strengths across shared SNPs (Figure S2).

## 4 Discussion

This study applied a bidirectional two‐sample MR analysis to systematically assess possible causal relationships between circulating inflammatory proteins, immune cells, and SAH. Our findings indicate that 5 inflammatory proteins and 22 immune cells exhibit causal associations with SAH. Specifically, CD6, TGFB‐1, and TGF‐α emerged as protective factors against SAH, whereas FGF23 and LIFR were identified as risk factors. Among the 22 immune cells, 10 were classified as risk factors and 12 as protective factors. Furthermore, reverse MR and Steiger analysis of the inflammatory proteins did not demonstrate a significant causal role. The MR Egger and MR‐PRESSO analyses confirmed no pleiotropy, supporting our main findings.

In the investigation of immune‐cell phenotypes, MR‐Egger regression indicated evidence of directional horizontal pleiotropy for the CD11b on CD33br HLA DR+ CD14dim myeloid cells, as reflected by a significant intercept. However, Cochran's *Q* test revealed no significant heterogeneity across instruments, and the MR‐PRESSO global test did not detect any outlier SNPs, suggesting that the observed pleiotropic signal is unlikely to be driven by a small number of extreme variants. Because MR‐Egger is sensitive to mild, systematic directional pleiotropy even in the absence of heterogeneity, this association should be interpreted with caution, and additional validation will be required. Furthermore, reverse MR analysis identified a possible connection between SAH and CD64 expression on CD14‐CD16+ monocytes. After SAH, diverse immune‐inflammation processes are triggered, associated with the production of inflammatory cytokines and immunomodulatory molecules. Our research discovered several inflammatory proteins and immune cells related to SAH. This research provides a novel understanding of the molecular processes involved in SAH and could pinpoint potential targets for future treatments.

FGF23, a crucial hormone in phosphate metabolism, may also affect iron metabolism, erythropoiesis, inflammation, insulin resistance, proteinuria, nephropathy, and left ventricular hypertrophy (Kanbay et al. 2017, Czaya and Faul 2019). Beyond its canonical role in phosphate metabolism, FGF23 has been shown to exert direct effects on vascular and inflammatory pathways. Elevated FGF23 levels can impair endothelial function by reducing nitric oxide bioavailability, increasing oxidative stress, and promoting vascular permeability, processes that are closely linked to cerebral aneurysm formation and rupture (Faul et al. 2011). In addition, dysregulated phosphate metabolism contributes to vascular stiffness and microvascular injury, potentially increasing susceptibility to SAH. Experimental studies further suggest that FGF23 can activate proinflammatory signaling pathways in endothelial and immune cells, providing biological plausibility for the observed causal association between FGF23 and SAH (Batko et al. 2019). Research indicates that the successful healing of intracranial aneurysms following endovascular treatment is contingent upon the maturation of early thrombus tissue and the formation of new intima, processes predominantly mediated by fibroblasts. In a model of large intracranial aneurysms, coiling treatment led to a significant elevation in FGF23 levels compared with untreated aneurysms, with a peak observed on day 7 (Grüter et al. 2023). A case‐control study demonstrated elevated FGF23 levels in cases of SAH relative to control subjects. In the general population, increased FGF23 levels were linked to an increased risk of SAH, regardless of other risk factors (Söderholm and Engström 2015). In addition to its biological and epidemiological relevance, FGF23 also represents a potentially druggable pathway. To explore this possibility, we evaluated 30 FGF23‐related drug candidates identified from DGIdb. Among these, cinacalcet—a clinically established calcimimetic—has been consistently shown to reduce circulating FGF23 levels in human studies, suggesting a plausible pharmacological route for modulating FGF23 signaling (Li et al. 2023). However, whether modifying FGF23 through cinacalcet or other related agents could influence outcomes in SAH remains unknown and requires systematic examination in mechanistic models and controlled preclinical studies.

Among the significant immunophenotypes identified, T‐cell and B‐cell phenotypes were predominant, comprising approximately 59% of the total. In contrast, monocyte and myeloid cell phenotypes constituted about 14%, dendritic cells accounted for approximately 9%, and natural killer (NK) cells represented about 4%. The essential involvement of immune cells in the pathophysiological processes of SAH is highlighted by this distribution pattern. A substantial amount of evidence indicates that the transient impairment of the immune system following aneurysmal SAH constitutes a significant risk factor for subsequent infections (Zhou et al. 2017). A prior clinical study identified an association between the risk of secondary SAH and immune‐mediated disorders (Ramagopalan et al. 2013). A decline in immune cell subsets, including CD3+, CD4+, CD8+ T cells, NK cells, and Treg cells, is observed postsurgery following SAH and is linked to poor prognosis (Jin et al. 2022). Our MR analysis indicates that Treg cells constitute 71% of the T‐cell population, highlighting their crucial role in the progression of SAH. Furthermore, our two‐step Mendelian randomization analysis demonstrated that CD4 regulatory T cells mediated approximately 10.8% of the genetically predicted effect of FGF23 on SAH, highlighting Tregs as a key immunological pathway linking FGF23 signaling to SAH susceptibility. Tregs are recognized for modulating the immune system by suppressing effector T cell proinflammatory responses (Arce‐Sillas et al. 2016). Recent studies show that Tregs infiltrate the brain soon after SAH, providing neuroprotection by secreting interleukin‐10 (IL‐10), which inhibits neuroinflammation and reduces neuronal apoptosis (Zhou et al. 2023). Another study demonstrated that IL‐2 therapy may attenuate neuroinflammation caused by SAH through the upregulation of the Treg population, thereby enhancing the long‐term prognosis of SAH (Dong et al. 2021).

Research on aneurysmal SAH patients' peripheral blood has revealed activation in immune cell subsets, including monocytes and neutrophils (Moraes et al. 2015). These findings underscore the role of innate immunity in SAH. The depletion of myeloid cells before the onset of SAH may reduce the incidence and severity of vasospasm following SAH (Provencio et al. 2011). His observation aligns with our MR results, which manifest that myeloid cells constitute a risk factor for SAH.

Building on previous research that associated immune cells with SAH (Yan and Li 2024), our study employed a bi‐directional two‐sample MR way to comprehensively evaluate the effects of these proteins and cells on SAH. In addition, we performed replication studies and colocalization analyses of important inflammatory proteins, which increased the reliability of our results. Although this study provides important insights, several limitations should be acknowledged. Even though we applied a stringent instrument‐selection threshold (*p* < 1×10^−5^) together with FDR correction, the large number of protein and immunophenotype comparisons inevitably increases the probability of false‐positive findings. This issue is further amplified by the correlation structure among immune cell traits, which may lead to dependent statistical tests. To mitigate these risks, we implemented a multilayer validation framework—including replication MR, colocalization, and multi‐omic convergence—to prioritize the most robust associations. In addition, the mediation analysis was conducted using summary‐level genetic data, which limited our ability to estimate uncertainty around the indirect effect using resampling‐based approaches such as bootstrap confidence intervals. As a result, the reported mediation proportion should be interpreted as an exploratory estimate rather than a precise quantitative effect. Furthermore, most datasets were derived from populations of European ancestry, potentially limiting generalizability to other ethnic groups, and partial sample overlap between GWAS datasets cannot be fully excluded. Future studies in diverse populations and with individual‐level data will be essential to validate and extend our findings.

## 5 Conclusion

This study suggests a causal link between inflammatory proteins and immune cells in SAH. Multi‐omics analyses, including transcriptome, colocalization, and mediated MR, identified FGF23 as a potential therapeutic target for SAH. These results emphasize the significant role of immunity and inflammation in SAH pathogenesis, enhance our understanding of its inflammatory and immune mechanisms, and uncover the intricate genetic causal relationships involved. Future studies are needed to further validate and expand on these findings.

## Author Contributions

**Xingjie Shi**: conceptualization. **Cheng Zhang**: conceptualization. **Tao Yang**: validation. **Zhiming Sun**: data curation, software. **Chao Wang**: writing – original draft. **Lucheng Zhou**: investigation. **Shiqiang Hou**: visualization, writing – review and editing. **Ning Lin**: funding acquisition, supervision. **Lanlan Zhang**: writing – review and editing.

## Funding

This research received funding from the Scientific Research Project of the Health Commission of Chuzhou (grant no. CZWJ2024A001), the Scientific Research Foundation of the Education Department of Anhui Province (grant no. 2024AH040093), and the Chuzhou Science and Technology Program (2024YF007).

## Conflicts of Interest

The authors declare no conflicts of interest.

## Supporting information




**Figure S1**: Instrument strength of inflammatory protein MR analyses. Distribution of per‐SNP F‐statistics for genetic instruments used in the Mendelian randomization analyses of inflammatory proteins. The dashed line indicates the conventional *F* = 10 threshold. All retained instruments exceeded this cutoff, indicating low risk of weak‐instrument bias.

**Figure S2**: Trans‐colocalization between FGF23 pQTL and SAH GWAS. Scatter plot showing concordant association signals between the SAH GWAS and FGF23 pQTL at the trans‐colocalization locus. Each point represents a shared SNP, with the lead variant rs3811621 highlighted. The pattern supports a shared causal variant (PPH4 = 0.74).

**Supplementary Tables**: brb371485‐sup‐0003‐TableS1‐S13.xlsx

## Data Availability

The data that support the findings of this study are available from the corresponding author upon reasonable request.
