# Supplementary figures and images for: FGF23 and Immune Cell Signatures Causally Linked to Subarachnoid Hemorrhage: Evidence From Multi‐Omics and Genetic Colocalization
Source: Brain Behav. 2026 May 8;16(5):e71485. doi: 10.1002/brb3.71485 (PMC13155464; doi:10.1002/brb3.71485)

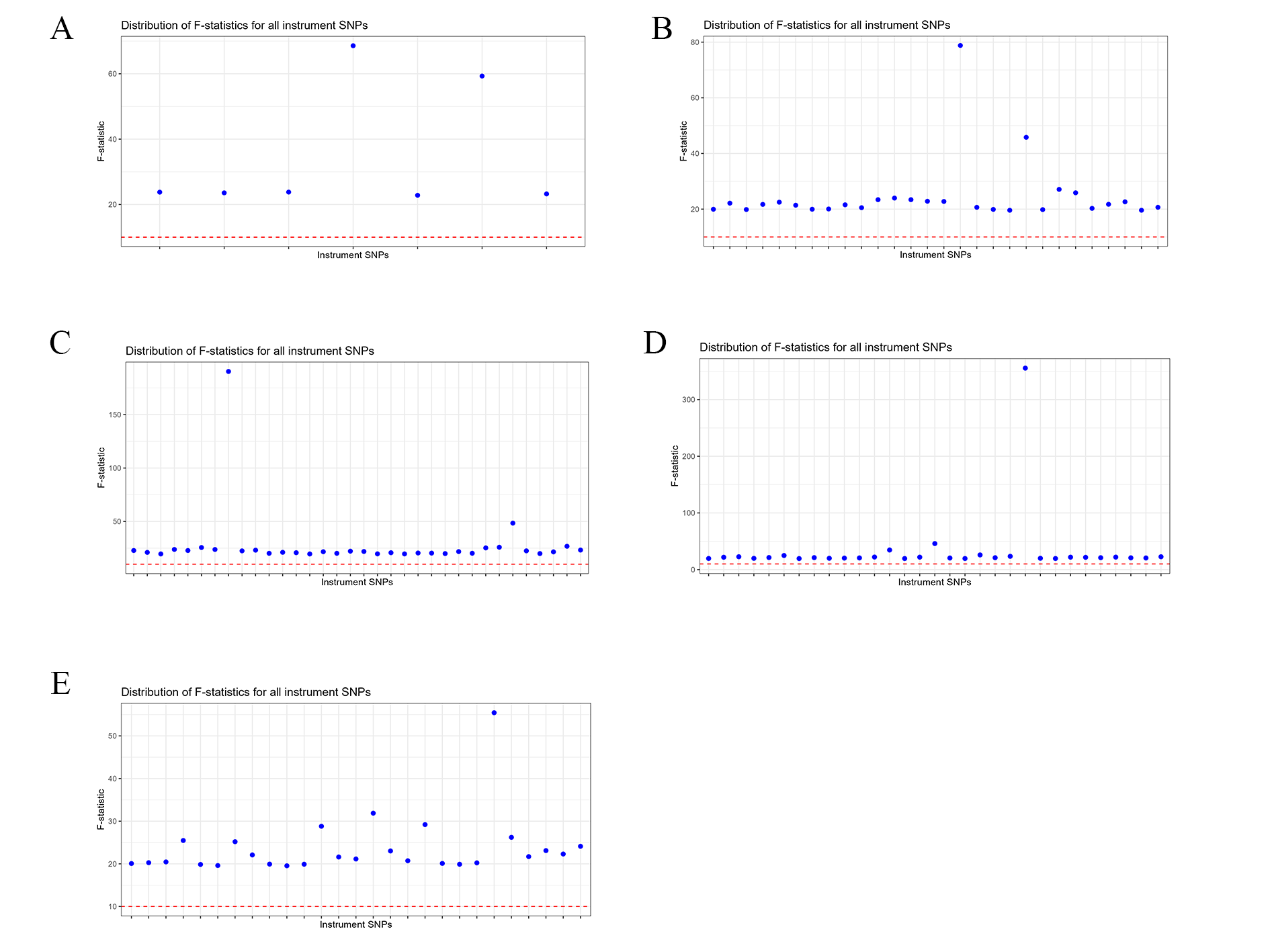

Supplement: Supplementary file 1 — Figure S1: Instrument strength of inflammatory protein MR analyses. Distribution of per‐SNP F‐statistics for genetic instruments used in the Mendelian randomization analyses of inflammatory proteins. The dashed line indicates the conventional F = 10 threshold. All retained instruments exceeded this cutoff, indicating low risk of weak‐instrument bias. [file BRB3-16-e71485-s003.tif]

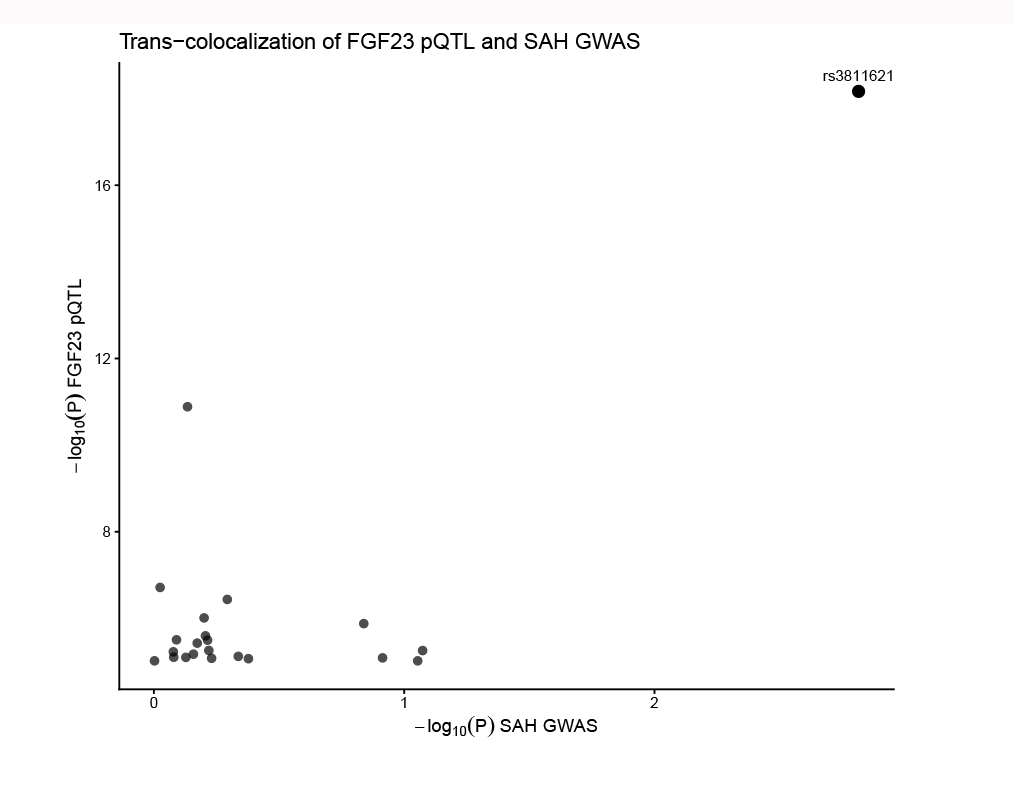

Supplement: Supplementary file 2 — Figure S2: Trans‐colocalization between FGF23 pQTL and SAH GWAS. Scatter plot showing concordant association signals between the SAH GWAS and FGF23 pQTL at the trans‐colocalization locus. Each point represents a shared SNP, with the lead variant rs3811621 highlighted. The pattern supports a shared causal variant (PPH4 = 0.74). [file BRB3-16-e71485-s002.tif]
